# Role of Exosomes in Islet Transplantation

**DOI:** 10.3389/fendo.2021.681600

**Published:** 2021-08-10

**Authors:** Jordan Mattke, Srividya Vasu, Carly M. Darden, Kenjiro Kumano, Michael C. Lawrence, Bashoo Naziruddin

**Affiliations:** ^1^Institute of Biomedical Studies, Baylor University, Waco, TX, United States; ^2^Islet Cell Laboratory, Baylor Scott and White Research Institute, Dallas, TX, United States; ^3^Baylor Simmons Transplant Institute, Baylor University Medical Center, Dallas, TX, United States

**Keywords:** exosome, biomarker, diabetes mellitus, miRNA, islet transplantation, islet stress, cytokines, nucleic acids

## Abstract

Exosomes are known for their ability to transport nucleic acid, lipid, and protein molecules, which allows for communication between cells and tissues. The cargo of the exosomes can have a variety of effects on a wide range of targets to mediate biological function. Pancreatic islet transplantation is a minimally invasive cell replacement therapy to prevent or reverse diabetes mellitus and is currently performed in patients with uncontrolled type 1 diabetes or chronic pancreatitis. Exosomes have become a focus in the field of islet transplantation for the study of diagnostic markers of islet cell viability and function. A growing list of miRNAs identified from exosomes collected during the process of isolating islets can be used as diagnostic biomarkers of islet stress and damage, leading to a better understanding of critical steps of the isolation procedure that can be improved to increase islet yield and quality. Exosomes have also been implicated as a possible contributor to islet graft rejection following transplantation, as they carry donor major histocompatibility complex molecules, which are then processed by recipient antigen-presenting cells and sensed by the recipient immune cells. Exosomes may find their way into the therapeutic realm of islet transplantation, as exosomes isolated from mesenchymal stem cells have shown promising results in early studies that have seen increased viability and functionality of isolated and grafted islets *in vitro* as well as *in vivo*. With the study of exosomes still in its infancy, continued research on the role of exosomes in islet transplantation will be paramount to understanding beta cell regeneration and improving long-term graft function.

## Introduction

Diabetes mellitus is characterized by chronic hyperglycemia due to partial or absolute insulin deficiency (type 1 diabetes, T1D) or pancreatic beta cell dysfunction and/or insulin inaction (type 2 diabetes, T2D) (1–3). In T1D, autoimmune cells infiltrate pancreatic islets (insulitis) and target pancreatic beta cells for destruction, leading to loss of the beta cell mass necessary for maintenance of euglycemia. T2D is a heterogeneous disease, with impairments or abnormalities in synthesis/secretion of insulin from islet beta cells, tissue sensitivity to insulin, or insulin action. Type 3c diabetes mellitus (T3cD), the least known or recognized form of diabetes mellitus (4, 5), is caused by pathologies of the exocrine pancreas that cause islet inflammation and damage (6). Chronic pancreatitis (CP), the most common cause of T3cD, is treated based on the etiology on a case-by-case basis. For refractory CP, partial or total pancreatectomy is performed to alleviate severe pain associated with chronic inflammation and necrosis. Current therapeutic options vary based on the type of diabetes. T2D is typically managed through lifestyle changes and medications targeting insulin resistance and/or beta cell function (7). For T1D and some cases of T3cD, exogenous insulin therapy is the standard of care. Even with aggressive therapy, a subset of diabetic patients present with glycemic lability, hypoglycemia unawareness, severe hypoglycemic episodes, diabetic ketoacidosis, and other complications of diabetes, including cardiovascular and kidney disease (8).

Pancreatic islet transplantation is an effective, minimally invasive treatment option for T1D or T3cD. In the 1980s, the first allo- (for T1D) and auto- (after pancreatectomy) islet transplants in humans were performed (9, 10), with poor long-term outcomes due to peri-transplant inflammation and ineffective immunosuppression (11, 12). In 2000, the Edmonton protocol with improved induction immunotherapy, a corticosteroid-free immunosuppressive regimen, and optimal islet dose, was established (13). Despite initial success in achieving insulin independence 1 year after transplantation, these patients exhibited poor long-term islet graft function. Several reports including our own data suggest that 50% to 70% of transplanted islet cells are lost during the islet isolation process and in the peri-transplant period due to an innate immune response called instant blood-mediated inflammatory reaction. This acute response involves the complement and coagulation systems and activation of inflammatory mediators (14, 15). Post-transplant inflammatory events lead to the recognition of the islet graft (either auto or allo) by the host immune system (innate or adaptive) and to the eventual rejection of transplanted islet cells. In allogeneic islet transplantation, alloantigen presentation to the host immune system triggers immune cell infiltration of the graft tissue, resulting in graft rejection. Observations in independent CP and T1D cohorts suggest that an optimal islet yield (>5000 islet equivalents/kg), islet survival in the peri-transplant period and engraftment, and optimal immunosuppressive regimen are important for achieving post-transplant insulin independence (16, 17). Despite the advancements made, short- and long-term graft survival and function remain suboptimal and a challenge to be overcome (18, 19).

Islet graft function and survival after transplantation are commonly assessed by glucose tolerance tests, C-peptide and hemoglobin A1c levels, exogenous insulin usage, and noninvasive imaging techniques (20, 21). However, these assessments tend to reflect the loss of islet function, offering little insight into islet stress, islet engraftment, and the ongoing loss of islet function. Establishing robust noninvasive methods to monitor islet survival and function after transplantation will help in the development of novel strategies to alleviate islet damage and improve transplantation outcomes. These challenges warrant further research on 1) delineating mechanisms of acute and chronic graft rejection, 2) monitoring islet stress and damage during and after transplantation, and 3) tailoring existing protocols to achieve improved short- and long-term outcomes.

In this regard, extracellular vesicles called ‘exosomes’ have emerged as prominent players in the assessment of islet function. Exosomes play an important role in donor-to-host cell communication, especially in presenting donor antigens to host immune cells and in horizontal transfer/dissemination of their content. Recent research also suggests a role for exosomes in carrying islet stress or damage molecular markers in circulation. In this review, we provide an overview of the roles of exosomes in allogeneic and autologous antigen presentation to the recipient immune system, exosome cargo, and the utility of exosomes for diagnosis and therapy.

## Exosome Formation and Release

Extracellular vesicles are released by almost all types of cells under physiological or pathophysiological conditions. These include microvesicles (100–1000 nm), apoptotic bodies (1–5 µm), oncosomes (1–10 µm), and exosomes (30–100 nm) (22, 23). Exosomes consist of a lipid bilayer membrane and an inner lumen containing diverse bioactive molecules including lipid, protein, and nucleic acid species (24, 25). After endocytosis, intraluminal vesicles (ILVs) are formed by inward budding of the endosomal membrane and are ultimately released as exosomes by exocytosis (26). These late endosomes containing ILVs are referred to as multivesicular bodies (27). During formation of ILVs, endosomal sorting complex required for transport (ESCRT) aids in the packaging of bioactive species, including nucleic acid species, proteins, and lipids in the ILVs. The first step in this process involves the ubiquitination of the cytoplasmic domain, which is recognized by ESCRT-0 (28) and then sequentially interacts with ESCRT-I, -II, -III to regulate cargo loading into the ILVs (29, 30). Another process for loading cargo into ILVs occurs independent of ESCRT, utilizing lipids such as lysobisphosphatidic acid and ceramides, exploiting the hydrophobic nature of lipids to bend the endosomal membrane inward to form vesicles (31). Following their formation, multivesicular bodies with ILVs may then be transported to lysosomes for degradation or fuse with the plasma membrane, resulting in exocytosis of the ILVs as exosomes and their contents (26). Circulating exosomes transfer their cargo between their parental and target cells, enabling cell-to-cell communication.

## Exosomal Protein Cargo in Islet Transplantation

Exosomes contain distinct nucleic acid and protein profiles reflecting the phenotypes of their cell source and cellular state. Exosomes are enriched in endosome-associated proteins including flotillins and annexins owing to their origin from endosomes. Exosomes also contain tetraspanins (CD9, CD81, CD82, CD37, and CD63), ESCRT proteins, heat shock proteins (HSC70 and HSC90), Alix, and TSG101 and are commonly used as exosome markers for research purposes (23).

Exosomes share surface major histocompatibility complex (MHC) antigens with their lineage. For example, dendritic cell–derived exosomes express CD80, CD86, and MHC class II molecules (32, 33). In a human-to-mouse xenogeneic islet transplant model and allogeneic human islet transplantation, transplanted human islets released donor human leukocyte antigen (HLA, MHC class I)–specific exosomes into the recipient circulation. Acute and long-term follow up of these recipients revealed gradual reduction in circulating donor HLA-specific exosomes, and elevated recipient T cell–derived CD3-specific exosomes, reflecting graft rejection (34). In both a mouse model and human allogeneic islet transplant recipients, donor HLA-specific exosomes contained islet endocrine hormones including insulin, glucagon, and somatostatin (protein and mRNA), which decreased after immune rejection of islet grafts (34). Exosomes derived from mouse insulinoma clonal cells (MIN6) expressed insulin and glutamic acid decarboxylase (GAD65) (T1D-associated autoantigen) in addition to exosome markers (35, 36). Ex vivo, human and rat islets released exosomes containing GAD65 and IA-2, autoantigens in T1D (37). In an allogeneic human islet transplant recipient, GAD65 antigen was detectable in donor HLA-specific exosomes at 455 days post-transplantation, with development of GAD65 autoantibodies at 1001 days post-transplantation (38). Our recently published observations revealed that human islets exposed to proinflammatory conditions released exosomes containing cytokines including IL-6, IL-8, MCP-1, and IP-10 (39). Proteomic profiling of MIN6-derived exosomes demonstrated enrichment of proteins involved in glycolysis, gluconeogenesis, citrate cycle, fructose and mannose metabolism, pyruvate metabolism, purine metabolism, and other metabolism-related pathways (35).

## Exosomal Nucleic Acid Cargo in Islet Transplantation and Its Utility as a Biomarker

**Figure 1:**
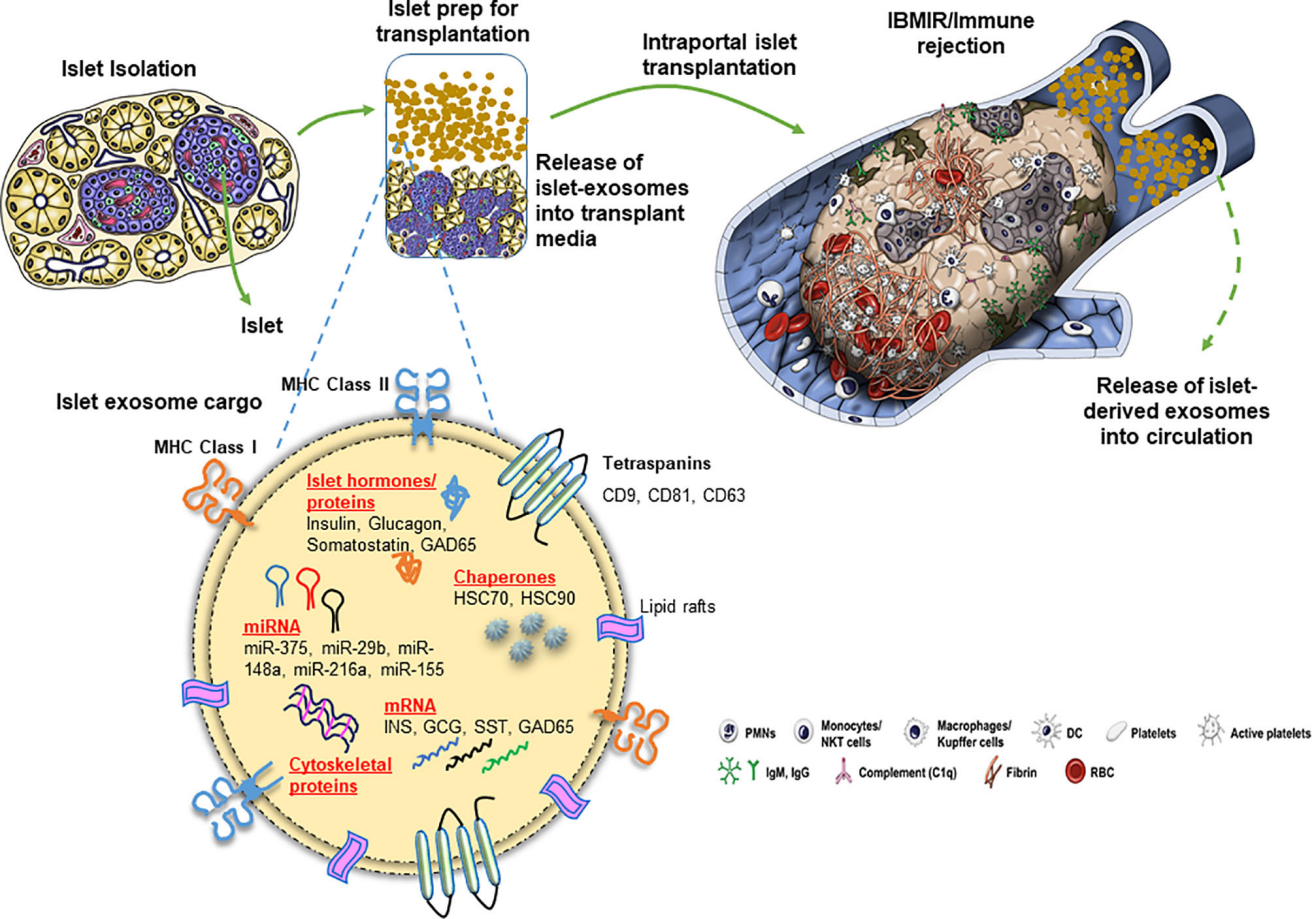
Release of exosomes and their cargo from human pancreatic islets during clinical islet transplantation.

**Figure 2:**
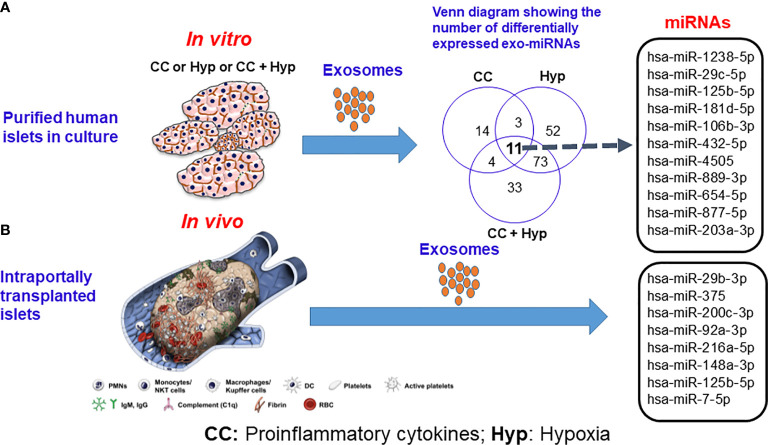
Release of exosomal microRNAs from human islets subjected to proinflammatory cytokines and hypoxic conditions **(A)** *in vitro* and **(B)** following intraportal transplantation of autologous islets *in vivo.* Source: Saravanan et al., *Diabetologia*, 2019 (36). CC indicates proinflammatory cytokines; Hyp, hypoxia.

Apart from proteins, intra-exosomal cargo contains nucleic acids, including DNA, mRNA, and miRNA. Loading of miRNA into exosomes is not a random process, as the types and diversity of miRNAs vary by cell type and cellular state at the time of sampling (40–43). Following intraportal transplantation, islets are exposed to hypoxic and inflammatory conditions, leading to loss of islet mass (**Figure 1**). *Ex vivo* mRNA and small RNA profiling of human islet–derived exosomes reveals distinct profiles depending on the islet culture conditions. Exosomes derived from human islets exposed to proinflammatory cytokines and/or hypoxia contain significantly higher levels of specific miRNAs. The Venn diagram in **Figure 2A** depicts the numbers of islet-derived exosomal miRNAs with differential expression under a) hypoxic, b) cytokine stress, and c) hypoxic and cytokine-induced cellular stress. Time course analysis revealed that miR-29b-3p and miR-216a-5p were released in exosomes starting 6 hours after hypoxia and cytokine exposure, relating to islet stress. miR-375 and miR-148a-3p were released in exosomes after 24 hours of hypoxic and cytokine-induced stress and damage, coinciding with onset of apoptosis (**Figure 2**). These early cellular stress and damage-induced exosomal miRNAs were also detected in plasma following islet transplantation in mice with streptozotocin-induced diabetes (43). During islet infusion and after transplantation in patients undergoing total pancreatectomy with islet autotransplantation, these miRNA markers were elevated in circulation, signaling islet stress and damage during transplantation and in the peri-transplant period (**Figure 1**) (43). In an independent study, human islets exposed to cytokines released exosomes containing 19 differentially expressed miRNAs, of which miR-155-5p, a well-known miRNA involved in inflammation, was the most upregulated miRNA (44). Apart from miRNAs, other small RNAs including piRNAs, lncRNAs, snoRNAs, and tRNAs were also identified in exosomes in this study (44). Among the small RNAs studied, exosomal miRNAs have potential as biomarkers of islet stress and damage in islet transplantation. Of particular interest are miR-375, miR-29b-3p, miR-148a-3p, miR-216a-5p, miR-200c-3p, miR-122-5p, miR-155-5p, and miR-221-3p, as these miRNAs have been identified consistently in an islet transplantation setting (43, 45). KEGG analysis revealed that miR-29b-3p, miR-216a-5p, miR-148a-3p and miR-375 target key molecules in PI3K/Akt, FOXO, mTOR signaling pathways and platelet activation (43). Further investigations are necessary to understand whether these islet stress and damage specific exosomal miRNAs alter key signaling pathways in immune cells including antigen presenting cells and lymphocytes in the context of islet transplantation.

In the context of diabetes, several studies have shown elevated levels of circulating exosomal miRNAs including miR-25-3p in T1D (46) and miR-125a-3p, miR-99b-5p, miR-197-3p, miR-22-3p, miR-27b-3p, miR-200a-3p, and miR-141-3p in gestational diabetes (47). Although a number of circulating miRNAs have been reported as elevated or reduced in circulation in T1D, T2D, obesity, and gestational diabetes, these studies were performed using plasma or serum fractions (45) and hence do not necessarily represent the exosomal miRNA content. Other types of extracellular vesicles including microvesicles may also contribute to the miRNAs in circulation.

Islet-derived exosomal mRNA cargo differs based on cellular state similar to exosomal miRNAs. After exposure to cytokines, 133 mRNAs were differentially expressed in human islet-derived exosomes. Although not differentially expressed, these human islet-derived exosomes also contained mRNAs coding for *IAPP, INS, MAFA, NEUROD1, NKX6.1, FOXO1, NEUROG3, PAX4*, and *SOX9* (44). In four allogeneic human islet transplant recipients, donor islet-specific exosomes contained insulin and glucagon mRNAs up to 1197 days after transplantation. One of these patients undergoing graft rejection also demonstrated circulating exosomes containing GAD65 mRNA at 455 and 1197 days after transplantation (38). In a human-to-mouse xenogeneic islet transplant model, donor HLA-specific exosomes contained mRNAs coding for insulin, glucagon, somatostatin, and FXYD2 (34). Thus, long-term graft function and survival can be monitored using circulating exosomal mRNA and endocrine hormones. Thus, non-invasive monitoring of islet graft function and survival by exosomal cargo using simple, non-invasive method supersedes existing diagnostic practices.

Although it is still unclear, exosomal cargo containing metabolism-, inflammation-, and cellular stress-related molecular species (**Table 1**) may exert distinct biological actions in target cells. In the context of islet transplantation, apart from their utility as biomarkers of islet stress and damage, transplanted islet-derived exosomes may serve as auto- or alloantigens triggering an immune response. Future anterograde tracing studies focusing on the actions of islet-derived exosomes on target cells in physiological and pathophysiological conditions are necessary. 

**Table 1:** Exosomal cargo in human islet transplantation.

Exosome content	Exosome source	Comments	References
hsa-miR-375	Culture supernatant	“Damage-induced exo-miRNA” increased in response to hypoxia, streptozotocin, and cytokine stress after 24 h	(36, 43)
	Xenotransplant mouse serum	Elevated 24 h following transplant	
	TPIAT supernatant/human Serum	Elevated throughout islet isolation	
hsa-miR-216a-5p	Culture supernatant	“Stress-induced exo-miRNA” increased after 6 h ctyokine and hypoxic stress	
	Xenotransplant mouse serum	Elevated 24 h following transplant	
	TPIAT supernatant/human serum	Elevated throughout islet isolation	
hsa-miR-148a-3p	Culture supernatant	“Damage-induced exo-miRNA” increased after 24 h cytokine and hypoxic stress	
	Xenotransplant mouse serum	Elevated 24 h following transplant	
	TPIAT supernatant/human serum	Elevated throughout islet isolation	
hsa-miR-29b-3p	Culture supernatant	“Stress-induced exo-miRNA” increased after 6 h ctyokine and hypoxic stress	
	Xenotransplant mouse serum	Elevated 24 h following transplant	
	TPIAT supernatant/human serum	Elevated throughout islet isolation	
hsa-miR-200c-3p	TPIAT supernatant/human serum	Elevated throughout islet isolation	
hsa-miR-3613-5p	Xenotransplant mouse serum	Increased in normoglycemic xenotransplant mice	(34)
Angiopoietin-1	Xenotransplant mouse serum	Increase associated with normoglycemia following xenotransplant	
HSC70	Xenotransplant mouse serum	Increase associated with normoglycemia following xenotransplant	
Complement C3	Xenotransplant mouse serum	Increase associated with rejection following xenotransplant	
Hemopexin	Xenotransplant mouse serum	Increase associated with rejection following xenotransplant	

TPIAT indicates total pancreatectomy with islet autotransplantation.

## Exosome Recognition by Immune Cells

Transplant rejection by the host immune system is a complex process, initiated by a series of events starting from recognition of the allograft or autoantigens, stimulation of the host’s immune system, and T cell–dependent rejection of the graft. Recent research has emphasized the roles of donor tissue–derived exosomes in recognition of the transplanted tissue by the host immune system. Purified allogeneic donor exosomes stimulate alloimmune responses by T cells *in vitro* and *in vivo*, emphasizing the significance of exosome recognition, uptake, and immune responses in transplantation (48). Exosomes weakly stimulate or fail to elicit immune responses in T cell lines and naïve T cells, respectively, and processing of exosomes by antigen-presenting cells (APCs) is required for T-cell activation (49–51).

After transplantation, recipient T cells can be stimulated through three pathways. The first is a *direct pathway*, where recipient T cells are stimulated by donor passenger APCs presenting an alloantigen on allogeneic MHC proteins (52). If the recipient T cells are naïve, donor passenger APCs must migrate to secondary lymphoid organs to induce T-cell responses. Structural differences in the donor MHC:alloantigen complex (multiple binary complexes hypothesis) (53) or each allo-MHC molecule on donor APCs (high-determinant density hypothesis) (54) may be recognized by T cells. The direct pathway is the driving mechanism behind acute graft rejection (55). The second pathway is the *indirect pathway*, where T cells recognize donor MHC/alloantigen-derived peptides processed and presented on self-MHC molecules by recipient APCs. T cells primed to recognize alloantigens are able to respond to peptides derived from allogeneic α and β chains of class II MHC molecules (56). Skin grafts from MHC II knockout mice were detected and rejected by MHC I knockout mice even though the recipient MHC I knockout mice lacked CD8^+^ T cells (57). The indirect pathway is important for alloantibody production and chronic rejection leading to graft vasculopathy and fibrosis (55). The third pathway is the *semidirect pathway:* Immediately after transplantation, donor passenger leukocytes do not travel to recipient regional lymph nodes for antigen presentation. Instead, recipient APCs take up donor exosomes containing donor MHC and antigens and process and present them on self-MHC molecules (cross-dressing), triggering T cell activation (32, 33, 55, 58). Recipient dendritic cells were able to acquire significant amounts of MHC molecules from both donor dendritic cells and endothelial cells (59). Graft rejection was evident in recipients who lacked the indirect allorecognition pathway (60). Following heart and islet transplantation in allogeneic mouse transplant models, recipient cells were cross-dressed with donor MHC antigens in draining and non-draining lymph nodes and spleen, with only a few passenger leukocytes at these sites (48). In a human-to-mouse xenogeneic islet transplant model, donor passenger leukocyte-derived exosomes were negligible in the donor HLA-specific exosome fraction, suggesting transplanted human islets as their source (34). Exosomes alone derived from rat mast cells only weakly stimulated specific T cells, and T-cell activation increased by fixation of exosomes to latex beads *in vitro* (49). MIN6-derived exosomes induced splenocytes isolated from NOD mice to produce proinflammatory cytokines including IL-6, IFN-γ, and TNF-α *via* the TLR-MyD88 signaling pathway. MIN6-derived exosomes increased CD86 expression on class II MHC-positive splenocytes and induced splenic T cell proliferation (36). After pro-inflammatory cytokine exposure, human islet-derived exosomes induced mRNA expression of NOS2 and COX2 in THP-1 cells, a macrophage cell line (39). The semidirect pathway is particularly important when recognizing donor peptides and MHC molecules by recipient immune cells. The involvement of the semidirect pathway in alloantigen presentation and stimulation of T cell responses is reviewed in detail elsewhere (55).

Thus, exosomes containing donor antigens, MHC molecules, and miRNAs can elicit immune responses directly (mostly negligible due to suboptimal levels of exosomal cargo to activate T cells) or indirectly through recipient APCs. In the context of islet autotransplantation, exosomes carrying islet stress and damage markers, sequestered self-antigens, and MHC molecules may be important in inducing autoimmune responses.

## Therapeutic Potential of Exosomes in Islet Transplantation

Of particular interest is induction of allogeneic tolerance or tolerance to sequestered self-antigens in autotransplantation. The immunogenicity of allogeneic or self-antigens depends on the dose, presentation site, other signals, and nature/state of APC activation. The ability of exosomes to induce immune tolerance is still unexplored, but exosomes as therapeutic delivery vehicles have been studied recently. Human bone marrow mesenchymal stem cells transfected with si-Fas and anti-miRNA-375 were co-cultured with peripheral blood mononuclear cells. Exosomes were collected from co-culture supernatant and transplanted into NOD-SCID gamma mice, resulting in decreased immune response and rejection by enhancing regulatory T cell function (61). In living donor liver transplant recipients, infusion of donor regulatory dendritic cells resulted in cross-dressing of recipient immune cells with surface expression of PD-L1, which was most likely transferred through donor exosomes containing PD-L1, CD73, and CD39. There was an increase in recipient regulatory CD4^+^ T cells as well as decreased activated CD8^+^ T cells after the infusion of donor regulatory dendritic cells (62).

More importantly, exosomes exhibit low toxicity, do not present a risk for tumor formation, and easily diffuse across biological barriers owing to their small size, which enables them to be used as injectable therapeutics (63). *In vitro*, mesenchymal stem cell–derived exosomes alleviated the detrimental effects of hypoxia-induced DNA damage, resulting in increased viability of porcine islet cell clusters in hypoxic conditions (64). Mouse islets cultured with mesenchymal stem cell–derived exosomes showed reduced expression of the pro-apoptotic genes *BAD* and *BAX* and increased expression of prosurvival genes *PI3K* and *BCL-2*, as well as increased production of vascular endothelial growth factor in mouse islets, resulting in increased production of insulin mRNA (65). Mesenchymal stem cell derived exosomes protected beta cells from apoptosis *via* the actions of miR-21 on ER stress and inhibition of phosphorylation of p38 (66). In streptozotocin diabetic mice, infusion of exosomes derived from bone marrow–derived mesenchymal stem cells induced regeneration of pancreatic islets (67). Administration of adipose tissue–derived exosomes into streptozotocin diabetic mice increased the regulatory T cell ratio in splenic mononuclear cells and improved insulitis (68). Exosomes isolated from lean adipose tissue explants also increased viability and functionality of isolated pancreatic β cells (69). These observations are proof of concept that stem-cell-derived exosomes may improve islet engraftment and functional outcomes of islet transplantation. Transplantation of MIN6-derived exosomes improved median survival time, glucose tolerance, insulin content, and islet architecture and reduced macrophage infiltration in streptozotocin diabetic mice (70). However, in diabetes-resistant NOR mice, immunization using MIN6-derived exosomes accelerated insulitis (36), highlighting that exosomes may either induce immune tolerance or induce a response to allogeneic or autologous antigens.

## Conclusions and Future Research Directions

Exosomes have emerged as important players in transplant rejection, as they carry donor antigens, which can weakly activate alloimmune responses through the direct or indirect allorecognition pathways. However, their major contribution to allorecognition comes in the form of cross-dressing recipient immune cells, leading to rejection of allogeneic transplanted tissue. Exosomal protein and nucleic acid cargoes also have potential for use as biomarkers for monitoring graft function and survival. Recent research highlights the utility of mesenchymal stem cell–derived exosome therapy during transplantation to improve islet survival and function, induce immune regulatory responses, and improve transplantation outcomes. Although exosome biology has been studied extensively, exosome research has its limitations. Exosome yield and quality vary between different isolation methods including ultracentrifugation, ultrafiltration and precipitation. Although exosomal miRNA, mRNA isolation and qPCR methods are robust, lack of exosomal housekeeping controls for normalization may influence experimental observations across laboratories. Additionally, there is no commercially available kit to measure very small amounts of exosomal miRNA/mRNA content accurately. Thus, there is a need for optimization and standardization of exosome research methods. Future studies should also be carried out using larger sample cohorts to validate and append the growing list of biomarkers for use in diagnostics. Exosomes in inducing transplant tolerance is an interesting area of research and may open up exciting and novel avenues in post-transplant immunosuppressive regimen. Given the contribution of islet-derived exosomes to graft rejection (34), future studies should focus on the fine line between induction of rejection and tolerance. With several studies demonstrating exosomes as safe therapeutic agents, continued studies into engineering and administration of exosomes in order to attenuate immune responses and prolonging graft survival will be of vital importance to the field. As exosome research is still in its infancy, its utility as biomarkers of islet stress and damage, inflammation or immune response and/or as therapeutics in the context of clinical islet transplantation should be validated and well-established independently across institutions.

## Author Contributions

JM, SV wrote the manuscript. All authors contributed to the article and approved the submitted version.

## Conflict of Interest

The authors declare that the research was conducted in the absence of any commercial or financial relationships that could be construed as a potential conflict of interest.

## Publisher’s Note

All claims expressed in this article are solely those of the authors and do not necessarily represent those of their affiliated organizations, or those of the publisher, the editors and the reviewers. Any product that may be evaluated in this article, or claim that may be made by its manufacturer, is not guaranteed or endorsed by the publisher.
